# Accurate and efficient gp120 V3 loop structure based models for the determination of HIV-1 co-receptor usage

**DOI:** 10.1186/1471-2105-11-494

**Published:** 2010-10-05

**Authors:** Majid Masso, Iosif I Vaisman

**Affiliations:** 1 Laboratory for Structural Bioinformatics, Department of Bioinformatics and Computational Biology, George Mason University, 10900 University Blvd. MS 5B3, Manassas, VA 20110, USA

## Abstract

**Background:**

HIV-1 targets human cells expressing both the CD4 receptor, which binds the viral envelope glycoprotein gp120, as well as either the CCR5 (R5) or CXCR4 (X4) co-receptors, which interact primarily with the third hypervariable loop (V3 loop) of gp120. Determination of HIV-1 affinity for either the R5 or X4 co-receptor on host cells facilitates the inclusion of co-receptor antagonists as a part of patient treatment strategies. A dataset of 1193 distinct gp120 V3 loop peptide sequences (989 R5-utilizing, 204 X4-capable) is utilized to train predictive classifiers based on implementations of random forest, support vector machine, boosted decision tree, and neural network machine learning algorithms. An *in silico *mutagenesis procedure employing multibody statistical potentials, computational geometry, and threading of variant V3 sequences onto an experimental structure, is used to generate a feature vector representation for each variant whose components measure environmental perturbations at corresponding structural positions.

**Results:**

Classifier performance is evaluated based on stratified 10-fold cross-validation, stratified dataset splits (2/3 training, 1/3 validation), and leave-one-out cross-validation. Best reported values of sensitivity (85%), specificity (100%), and precision (98%) for predicting X4-capable HIV-1 virus, overall accuracy (97%), Matthew's correlation coefficient (89%), balanced error rate (0.08), and ROC area (0.97) all reach critical thresholds, suggesting that the models outperform six other state-of-the-art methods and come closer to competing with phenotype assays.

**Conclusions:**

The trained classifiers provide instantaneous and reliable predictions regarding HIV-1 co-receptor usage, requiring only translated V3 loop genotypes as input. Furthermore, the novelty of these computational mutagenesis based predictor attributes distinguishes the models as orthogonal and complementary to previous methods that utilize sequence, structure, and/or evolutionary information. The classifiers are available online at http://proteins.gmu.edu/automute.

## Background

Host cells targeted for entry by HIV-1 express the cellular CD4 receptor as well as a secondary cellular chemokine co-receptor, principally either CCR5 (R5) or CXCR4 (X4), all of which interact with the HIV-1 envelope glycoprotein gp120. Natural ligands for these receptors include IL-16 (CD4); RANTES, MIP-1α, and MIP-1β (R5); and SDF-1α (X4) [1]. Prior to being recognized that successful viral entry necessarily requires that gp120 also binds a co-receptor subsequent to CD4 attachment, HIV-1 strains were typically classified as nonsyncytium (NSI)- or syncytium (SI)-inducing based solely on their ability to induce syncytia in cell cultures, which correlates with viral preference for infecting monocyte-derived macrophages (M-tropic) or T-lymphocytes (T-tropic), respectively [2,3]. M-tropic strains of HIV-1 often use the R5 co-receptor while T-tropic strains use X4; however, there also exist dual- or mixed-tropic (DM or R5/X4) strains capable of using both co-receptors [4].

**Figure 1:**
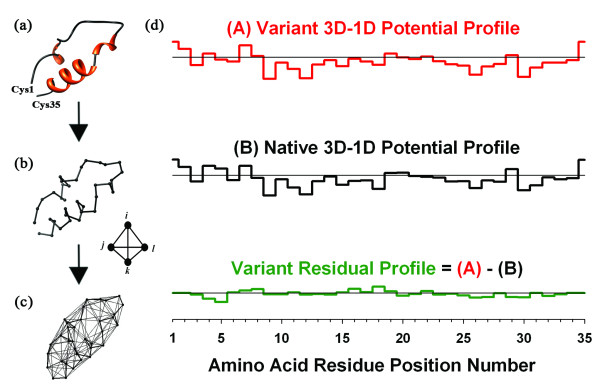
**HIV-1 gp120 V3 loop structure visualization, Delaunay tessellation, and variant representation**. (a) Ribbon diagram and (b) C_α _trace of the V3 loop peptide structure (PDB ID: 1ce4, NMR model 1). (c) Delaunay tessellation of the V3 loop superimposed over the trace. (d) 3D-1D potential profiles for both the native R5-tropic V3 loop sequence corresponding to PDB structure file 1ce4, as well as an R5-tropic variant V3 loop sequence defined by the five substitutions N5G, H13R, R18Q, T22A, and I26V threaded onto the native structure tessellation. The variant residual profile is defined as the difference between the variant and native 3D-1D potential profiles and measures environmental perturbations at all 35 V3 loop positions due to the substitutions.

The significance of viral categorization based on co-receptor usage is underscored by the observation that while a majority of newly infected patients harbor only R5-utilizing HIV-1 strains, X4 variants appear in approximately 50% of patients during later stages of the disease accompanied by an accelerated decline in CD4+ T-lymphocytes and progression towards an AIDS diagnosis [5]. Co-receptors R5 and X4 interact to a great extent with the third hypervariable loop (V3 loop) of the HIV-1 envelope glycoprotein gp120 [6], a peptide fragment distant from the gp120 core and comprised of 35 amino acids with a disulfide bridge formed by cysteine residues at the N- and C-termini (Fig. 1). This interaction suggests that accumulation of amino acid replacements at multiple positions within the V3 loop is responsible for the eventual switch in co-receptor affinity; however, there are competing arguments as to whether V3 loop structural changes drive co-receptor selectivity, or if one predominant conformation exists for both R5 and X4 variants and that sequence changes alone account for the switch in co-receptor usage [7,8]. Evidence suggesting a dual contribution was provided by a study in which knowledge-based potentials were used to assess the fitness of variant V3 loop sequences on candidate structures generated by Markov Chain Monte Carlo techniques applied to NMR data [9].

Widespread clinical success through HIV-1 combination drug therapy, targeting essential proteins at distinct stages of the viral life cycle, is tempered by the emergence of patient viral strains that are resistant to one or more of these medications. Hence the identification of healthy individuals, homozygous for a nonfunctional R5 due to a 32-base pair deletion and highly resistant to HIV-1 infection [10], motivated the search for viable co-receptor antagonists to include among the current arsenal of treatments. The US Food and Drug Administration recently approved the R5 inhibitor maraviroc [11] for use in treatment-experienced individuals in combination with other medications, and a number of additional co-receptor antagonists are in various stages of development [12,13]. Evaluation of viral co-receptor tropism in patients is essential prior to and during clinical administration of such antagonists so that only the appropriate co-receptor is targeted, and *in vitro *experimental assays are available; however, these phenotype tests are costly and time-consuming, and their results are not standardized [14]. Given these drawbacks, accurate *in silico *models for predicting co-receptor tropism have generated considerable interest. The vast majority of these methods rely on patient HIV-1 gp120 V3 loop sequences easily obtained through relatively rapid and inexpensive genotype tests, which are predicted to be either strictly R5-tropic or X4-capable (i.e., X4 and R5/X4 combined), since there are relatively far fewer sequences that strictly use X4. For the sake of notational simplicity, the DM acronym is modified in the remainder of this manuscript to refer to X4-capable virus, yielding two co-receptor usage categories: R5 and DM.

The 11/25 charge rule refers to the earliest and simplest predictive method reported [15], identifying HIV-1 with an SI phenotype based on the presence of positively charged residues at V3 loop positions 11 and/or 25 with good specificity though relatively poor sensitivity with respect to DM strains, the majority of which induce syncytia [16,17]. A second approach utilized a sequence based multiple linear regression and identified four positive predictors of HIV-1 co-receptor tropism: the number of positively (K/R) and negatively (D/E) charged V3 sequence residues, the V3 net charge (K/R - D/E), and the presence of I at gp120 position 292 outside of the V3 loop (V3 loop begins at C296) [18]. Next, predictions of HIV-1 phenotype and co-receptor usage have been obtained through position-specific scoring matrix (PSSM) techniques [6,19,20]. Finally, several models have been developed recently based on a variety of supervised classification machine learning algorithms utilizing V3 sequence and/or structure descriptors. These algorithms have included neural network (NN) [17,20], decision tree (DT) [20-22], support vector machine (SVM) [20-23], and random forest (RF) [22,24]. The addition of structural attributes to the sequence features of V3 loop variants was shown to significantly improve classifier prediction performance [23].

In this article, we describe a computational mutagenesis methodology for characterizing HIV-1 gp120 V3 loop variants that involves threading of sequence (translated genotype) information onto a reference structural template and relies on a four-body, knowledge-based potential (Fig.1). For each V3 loop mutational pattern, the approach yields both a scalar measure of overall change in sequence-structure compatibility relative to the native peptide, as well as a 35-dimensional vector representing environmental perturbations at all V3 loop residue positions caused by the amino acid replacements. In particular, we demonstrate that sequence-structure compatibility is more adversely affected among DM-tropic strains relative to R5 variants in a non-redundant dataset of 1193 V3 loop sequences with known co-receptor tropism. Additionally, with each sequence represented as a perturbation vector, the dataset is used for training a variety of machine learning algorithms. This novel approach utilizing both sequence and structure, as well as combining machine learning with an energy function-based mutagenesis for mutant representation, was previously applied to develop accurate models for predicting susceptibility to HIV-1 protease inhibitors [25]. The classifiers developed here are shown to outperform other published models for V3 loop-based prediction of co-receptor usage, especially with regard to the sensitivity of DM strain classification, suggesting that signals inherent in these vectors are more effective for discrimination between R5 and DM viral strains. These models are also well suited to provide instantaneous predictions and require only V3 loop sequences as input.

## Methods

### Dataset

A search of the HIV Sequence Database at Los Alamos National Laboratory (http://www.hiv.lanl.gov/content/sequence/HIV/mainpage.html, accessed in April 2009) generated a total of 3986 HIV-1 gp120 V3 loop sequences with annotated co-receptor phenotypes obtained from treatment naïve and experienced patients (3693 sequences associated with 738 distinct patients, and 293 sequences with missing patient ID numbers), spanning all HIV-1 subtypes and based on data obtained from clinical trial studies in 39 countries over sampling years 1983-2006. Upon translation and elimination of duplicate sequences, the final dataset contained 1193 distinct 35-residue long V3 loop sequences, consisting of 989 strictly R5-utilizing variants and 204 DM variants. In order to be consistent with all other methods to which we compare our results, and due to the paucity of strictly X4 variants in the dataset, by convention the DM category here more generally consists of X4-capable sequences (i.e., both X4 and R5/X4 variants are combined).

### Computational mutagenesis methodology

Our *in silico *mutagenesis procedure relies on a four-body, knowledge-based, statistical contact potential, which provides an interaction empirical energy for each of the 8855 permutation-free quadruplets of amino acids that can be enumerated using a standard 20-letter protein alphabet [26]. We generated the four-body potential by analyzing a diverse dataset of 1417 high-resolution (≤ 2.2Å) crystallographic protein structures with low sequence identity (< 30%), culled from the Protein Data Bank (PDB) [27] using the PISCES server [28]. The C_α _atomic coordinates of the constituent amino acid residues were used to render each protein structure as a discrete collection of points in 3-dimensional (3D) space. Delaunay tessellation, a classical computational geometry technique, was used to model each protein, whereby C_α _atoms served as vertices to generate a 3D convex hull formed by a tiling of non-overlapping, irregular, tetrahedral simplices [29]. Hundreds of simplices are generated by the tessellation of an average sized protein, and the four vertices of each simplex objectively identify a quadruplet of nearest neighbor amino acid residues in the protein structure. For each of the 8855 amino acid quadruplets (*i*, *j*, *k*, *l*), a relative frequency of occurrence *f*_*ijkl *_was calculated based on the proportion of simplices among the 1417 protein structure tessellations whose vertices represent the four residues, and a rate of quadruplet occurrence expected by chance *p*_*ijkl *_was obtained from a multinomial reference distribution [26]. Energy of quadruplet interaction, modeled after the inverse Boltzmann principle, was calculated as log(*f*_*ijkl*_/*p*_*ijkl*_) [26,30].

Next, Delaunay tessellation was applied to a V3 loop structure (PDB ID: 1ce4, model 1) containing an R5-tropic sequence (JRCSF isolate), which is currently the only available structure of the unliganded peptide [31]. Using the four-body potential and tessellated V3 loop (Fig. 1) [31,32], each of the constituent simplices was scored according to the interaction energy of the amino acid quadruplet represented by its vertices. A total of 130 tetrahedral simplices were generated by the V3 loop tessellation, where all six edges for 105 simplices have lengths less than 12Å, and the longest edge among the remaining 25 simplices measures 20.93Å. The global sum of the 130 simplex scores, referred to as the V3 loop *total potential *or *topological score*, provides an overall sequence-structure compatibility measure for the native peptide [33-35]. For each V3 loop amino acid, an *individual residue potential *or *residue environment score *was calculated as the local sum of all simplices that share the C_α _atom of the amino acid as a vertex [34-37], and an ordered representation of these scores by primary sequence position number as a 35-dimensional vector forms a *3D-1D potential profile *(Fig.1) [38].

For each particular V3 loop mutational pattern, the new sequence of amino acid letters was threaded onto the native tessellated V3 loop template structure by relabeling each of the 35 C_α _vertices. While the tessellation remains unaffected, simplices with one or more relabeled vertices are recast with altered scores due to new residue quadruplet compositions, and recalculations yield a topological score and 3D-1D potential profile for the V3 loop variant. The difference between variant and native V3 loop topological scores, referred to as the variant *residual score*, measures relative change in overall sequence-structure compatibility due to the residue substitutions [34-37]. Finally, the variant *residual profile *is defined as the difference between the variant and native V3 loop 3D-1D potential profiles and consists of component values measuring environmental perturbations at all V3 loop positions (Fig. 1) [35-37]. Conformational changes in the protein structure are effectively taken into account with this computational mutagenesis methodology, both implicity, through the four-body potential and variant residual scores and profiles, and explicitly, due to the use of only coarse-grained C_α _representations of protein structures and the fact that Delaunay tessellation is robust to small shifts in the C_α _coordinates [34-37].

### Machine learning tools for prediction and evaluation of performance

The dataset of 1193 HIV-1 gp120 V3 loop variants was used to train and compare the performance of four well-known supervised learning algorithms, random forest (RF), support vector machine (SVM), boosted decision tree (BDT), and neural network (NN), all available as part of the Weka software package [39]. Residual profiles were utilized as input feature vectors for characterizing V3 loop variants, and variant co-receptor tropism (R5 or DM) represented the output attribute for the classifiers. Non-default values of the adjustable parameters used in the implementation of these algorithms include: one hundred bootstrapped datasets (i.e., one hundred classification trees for majority vote) for RF; radial basis function (RBF) kernel with γ = 0.1, neither normalization nor standardization of the training data, and logistic models fit to the outputs for SVM; 50 boosted iterations using the Adaboost M1 method for BDT; and no attribute normalization for NN.

Algorithm performance on the dataset was evaluated by using stratified 10-fold cross-validation (10-fold CV), leave-one-out cross-validation (LOOCV), and stratified random split (66% of the dataset used for model training and the remaining 34% used for prediction) testing procedures. Prediction results reported with 10-fold CV and 66/34 split are based on averages over ten independent iterations.

Assuming P (positive) refers to the DM class and N (negative) refers to the R5 class, ACC = accuracy = (TP + TN)/(TP + FN + FP + TN) provides a simple measure of the overall prediction success rate. Here, TP and TN represent the number of correct DM and R5 predictions, respectively, and FP and FN are misclassifications. The balanced error (BER) and balanced accuracy (BAR) rates, calculated as BER = 0.5 × [FN/(FN + TP) + FP/(FP + TN)] and BAR = 1 - BER, Matthew's correlation coefficient (MCC), given by

$$\text{MCC}=\frac{\text{TP}\times \text{TN-FP}\times \text{FN}}{\sqrt{\text{(TP}+\text{FN)(TP}+\text{FP)(TN}+\text{FN)(TN}+\text{FP)}}},$$

and area (AUC) under the receiver operating characteristic (ROC) curve provide additional measures of classifier performance that are especially useful for unbalanced class distributions. The ROC curve is a plot of the true positive rate (sensitivity) versus the false positive rate (1 - specificity), where sensitivity = Se(DM) = TP/(TP + FN) and specificity = Sp(DM) = TN/(TN + FP). An AUC value near 0.5 is indicative of random guessing while a value of 1.0 denotes a perfect classifier. Finally, positive predictive value or precision is defined as PPV(DM) = TP/(TP + FP).

## Results and Discussion

### Variant V3 loop dataset sequence analysis

**Figure 2:**
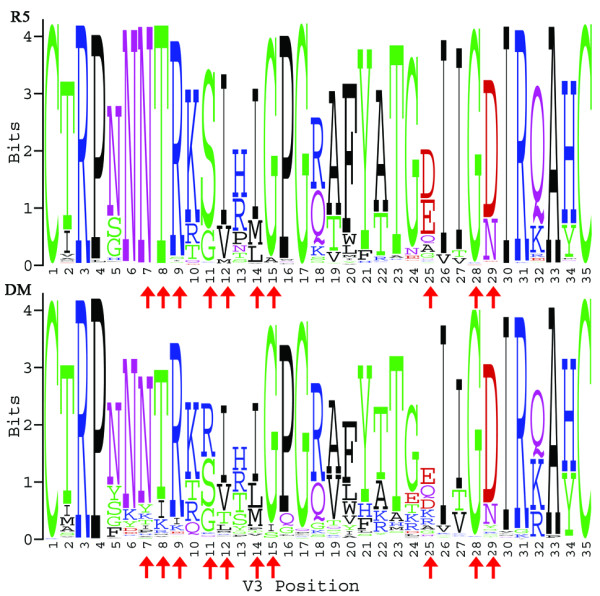
**V3 loop sequence logos for the 989 R5 and 204 DM dataset variants**. The size of the letters at each position is indicative of their relative frequencies of occurrence among the sequences in the co-receptor class. The red arrows identify the top ten positions in the variant residual profile vectors for predicting co-receptor phenotype, ranked by an SVM classifier and a 10-fold CV attribute selection mode.

**Figure 3:**
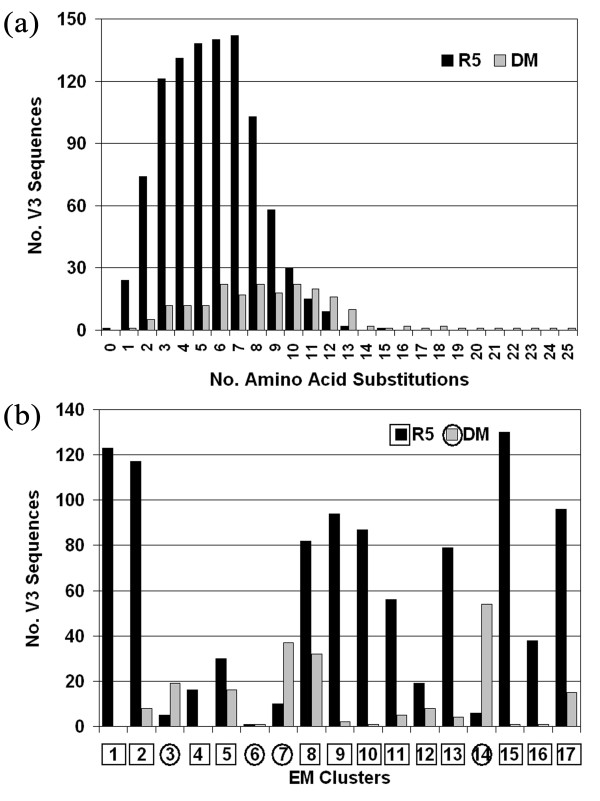
**Data analysis and clustering of V3 loop variant sequences**. (a) Histograms for the sets of R5 and DM V3 loop variant sequences based on the number of residue substitutions relative to the native sequence of PDB structure 1ce4. (b) Unsupervised EM clustering of the variant V3 loop sequence dataset based on their residual profile representations yields a cluster purity measure of 90.4%.

For each co-receptor usage class, variant HIV-1 gp120 V3 loop sequences were aligned and sequence logos [40] were generated to visualize relative amino acid frequencies at each position and to identify highly conserved positions (Fig. 2). Similarities (e.g., highly conserved cysteine residues at terminal positions) and differences (e.g., amino acid relative frequencies at positions 11/25) are clearly evident between both logos. Additionally, histograms were produced for each co-receptor class based on the number of amino acid substitutions in the variant V3 loop sequences relative to the native sequence of the tessellated structure (Fig. 3a). The average number of V3 loop residue replacements was calculated as 5.6 ± 2.4 for the R5 class and 8.8 ± 4.2 for the DM class, and a *t*-test revealed a statistically significant difference between class mutation averages (*p *< 0.0001). These data suggest that a greater accumulation of mutations is associated with a switch in co-receptor affinity, which may be due to either V3 loop conformational changes or stochastic accumulation of minor mutations prior to co-receptor switch substitutions at sequence positions 11/25.

Based on an analysis of variant V3 loop changes in sequence-structure compatibility relative to the native peptide (i.e., the variant residual scores), stark differences also exist between the R5 and DM co-receptor classes. The mean residual score was calculated to be 0.32 for the 989 V3 loop variants comprising the R5 class and -0.60 for the 204 DM-tropic variants, and a *t*-test revealed a statistically significant difference between class mean residual scores (*p *< 0.0001). These results strongly suggest that the continual accumulation of mutations in the DM class is ultimately detrimental to V3 loop sequence-structure compatibility.

Finally, with each V3 loop variant represented by its respective 35-dimensional residual profile vector of environmental perturbation scores, an unsupervised clustering of the variants was performed using the expectation-maximization (EM) algorithm available with the Weka software package [39]. For each variant, the EM algorithm calculates probabilities of membership in each of the available clusters, and the algorithm uses a cross-validation procedure to automatically determine the number of clusters. A total of 17 clusters were generated, each labeled as either R5 or DM based on majority class size (Fig. 3b). Cluster purity, defined as

$$\text{Purity}=\sum_{j}\frac{n_{j}}{n}{\text{ max}}_{i}\left\{\text{precision}\left(i,j\right)\right\}$$

where precision (*i*, *j*) refers to the relative frequency of class *i *in cluster *j*, *n*_*j *_is the number of sequences in cluster *j*, and *n *= 1193 is the dataset size, is 90.4%. The clustering can be associated with a guiding tree that reflects the distances between V3 loops in a sequence space and likely corresponds to the evolutionary history of the virus. Thus, given a training set of V3 sequences collected from the same set of patients at multiple time points, starting from early stages of infection, it would be possible to build a model to correlate V3 sequence position in a tree and likelihood of a switch in co-receptor usage.

### Predictive performance of variant V3 loop residual profiles

**Figure 4:**
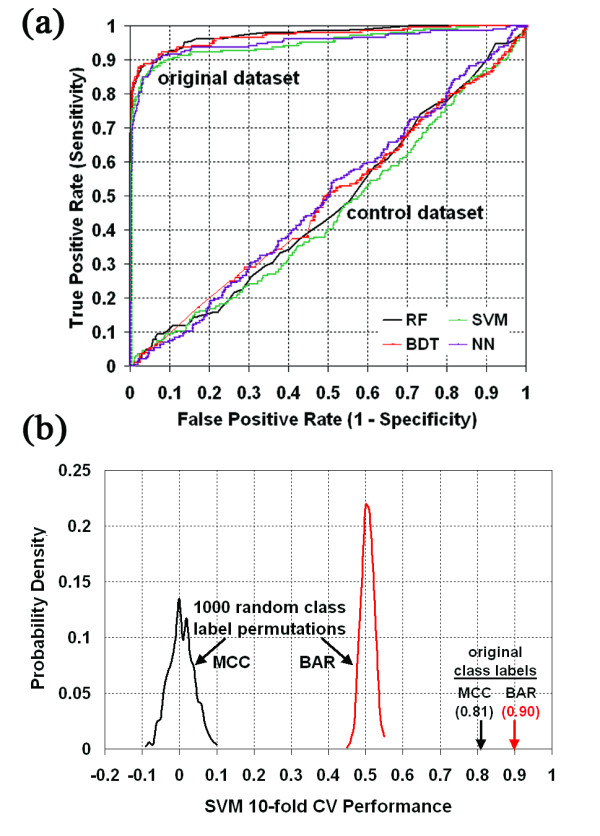
**Statistical significance of classifier performance**. (a) LOOCV ROC curves for all four models based on the original dataset as well as a control obtained via a single random permutation of the R5/DM class labels among the V3 loop variants in the dataset. (b) Distribution of 10-fold CV SVM prediction performance (BAR - balanced accuracy rate and MCC - Matthew's correlation coefficient) over 1,000 permutations (random shuffles) of the class labels, compared with similar measurements obtained using the class label arrangement of the original dataset.

Four supervised classification algorithms (RF, SVM, BDT, and NN) were utilized for assessing predictive performance of the environmental perturbation descriptors encoded by the variant V3 loop residual profile vectors, and results were reported based on application of 10-fold CV, LOOCV, and 66/34 split testing procedures (Table 1). The testing methods and algorithms all generated relatively consistent results with the exception of slightly lower values based on NN. In order to highlight the strength of signals embedded in the variant V3 loop residual profiles for accurately discriminating between R5-utilizing and DM (X4-capable) sequences, we compared the LOOCV results of Table 1 using the original dataset with those obtained using a control dataset generated by a randomly shuffling of the R5 and DM class labels among the V3 loop variants so that the class sizes are unaltered. Striking AUC reductions to levels near 0.5 were observed using the control dataset (Fig. 4a), suggesting that models developed with the control are not likely to perform better than random guessing, a conclusion further supported by MCC and BER measures: RF (MCC = 0.01, BER = 0.49), SVM (MCC = -0.02, BER = 0.51), BDT (MCC = -0.01, BER = 0.50), and NN (MCC = -0.02, BER = 0.51). For a more systematic approach to assessing the statistical significance of our results in Table 1, we generated 1,000 class label permutations (random shuffles) and calculated 10-fold CV performance in each case based on the SVM algorithm. The distributions of MCC and BAR accuracy measurements (Fig. 4b) are narrowly centered around zero and 0.5, respectively (MCC = 0.00 ± 0.03, BAR = 0.50 ± 0.02), with no permutation accuracy approaching those obtained using the original arrangement of the class labels (Table 1: MCC = 0.81 ± 0.01, BAR = 1 - BER = 0.90 ± 0.01), so that the *p*-value for predictive power is less than 0.001. Results nearly identical to these were also obtained using the RF (MCC = 0.00 ± 0.03, BAR = 0.50 ± 0.01), BDT (MCC = 0.00 ± 0.03, BAR = 0.50 ± 0.01), and NN (MCC = 0.00 ± 0.03, BAR = 0.50 ± 0.02) algorithms, whereby 10-fold CV was applied to each of 1,000 new random class label shuffles generated for each of the three methods, and these data can be compared to the 10-fold CV performance for each of the algorithms based on the original class label arrangement as provided in Table 1.

**Table 1:** Comparison of performance measures

Method	ACC	Se(DM)	Sp(DM)	PPV(DM)	AUC	MCC	BER
**RF (LOOCV)**	0.96	0.82	0.99	0.95	0.97	0.86	0.09
**RF (10-fold CV) **^**a**^	0.96	0.82	0.99	0.95	0.97	0.87	0.09
**RF (66/34 split) **^**a**^	0.96	0.84	0.99	0.94	0.97	0.87	0.08
**SVM (LOOCV)**	0.95	0.84	0.97	0.87	0.95	0.82	0.09
**SVM (10-fold CV) **^**a**^	0.95	0.83	0.97	0.86	0.95	0.81	0.10
**SVM (66/34 split) **^**a**^	0.95	0.85	0.97	0.84	0.96	0.81	0.09
**BDT (LOOCV)**	0.96	0.82	0.99	0.97	0.97	0.87	0.09
**BDT (10-fold CV) **^**a**^	0.96	0.80	0.99	0.96	0.97	0.85	0.10
**BDT (66/34 split) **^**a**^	0.97	0.83	1.00	0.98	0.97	0.89	0.08
**NN (LOOCV)**	0.95	0.80	0.98	0.87	0.95	0.80	0.11
**NN (10-fold CV) **^**a**^	0.95	0.80	0.98	0.86	0.95	0.80	0.11
**NN (66/34 split) **^**a**^	0.94	0.82	0.97	0.83	0.95	0.79	0.11
**Sander *et al. *** **(SVM, 10-fold CV) **^**a**^	0.92	0.80	0.95	0.81	0.93	-----	-----
**Prosperi *et al. *** **(RF, 10-fold CV) **^**a**^	0.88	0.63	-----	-----	0.88	-----	-----
**Prosperi *et al. *** **(SVM, 10-fold CV) **^**a**^	0.90	0.69	-----	-----	0.91	-----	-----
**Sing *et al. *** **(SVM, 10-fold CV) **^**a**^	-----	0.76	0.93	-----	-----	-----	-----
**Xu *et al. *** **(RF, 56/42 split) **^**b**^	0.95	0.85	0.98	0.99	-----	0.87	-----
**Pillai *et al. *** **(SVM, 10-fold CV) **^**c**^	0.91	0.76	0.98	0.95	-----	-----	-----
**Resch *et al. *** **(NN, 50/50 split) **^**c**^	-----	0.75	0.94	0.69	-----	-----	-----

^**a **^average over 10 iterations; ^**b**^single split - use of duplicates inflates performance; ^**c**^average over 100 iterations

Our sequence-structure approach, combining machine learning with an energy-based computational mutagenesis for generating variant V3 loop sequence feature vectors, outperformed the structure-based approach of Sander *et al. *[23] and the sequence-based approaches of Prosperi *et al. *[22], Sing *et al. *[20], Xu *et al. *[24], Pillai *et al. *[21], and Resch *et al. *[17] (Table 1). These studies all obtained datasets from the Los Alamos HIV Sequence Database in an approach similar to the one outlined in this manuscript. Sander *et al. *and Sing *et al. *utilized the SVM algorithm along with a 10-fold CV testing procedure, Prosperi *et al. *used the RF and SVM algorithms with 10-fold CV, and the results of all three groups reflect an average over ten iterations. Xu *et al. *applied the RF algorithm with only a single 56/44 random split testing approach; moreover, the full dataset contained an abundance of duplicates that were not removed prior to the split. While duplicates within the test set were subsequently removed prior to evaluating performance, duplicates between the training and test sets were not addressed, which would artificially inflate the reported performance. Pillai *et al. *utilized the SVM algorithm and averaged the results over 100 iterations of 10-fold CV. Lastly, Resch *et al*. implemented an NN algorithm and reported results based on 100 iterations of 50/50 random splits.

The practical value of these models is measured by their ability to reliably predict co-receptor usage classes for V3 sequences not appearing in the training dataset, therefore constituting an independent test set. Yet obtaining and annotating additional testing samples in a timely way is often not feasible. Alternatively, stratified random split of an annotated dataset of distinct V3 sequences into one subset for training and another for testing, such as those two-subset splits whose results are reported in Table 1, accurately reflect the performance expected by trained models on independent test sets. In a recent study, Low *et al. *[16] evaluated six algorithms with a test set of 920 V3 sequences whose co-receptor usage annotations are not available in the public databases. They reported Se(DM) values in the 0.24 - 0.50 range, discounting utility of the models. However, these difficulties were likely encountered because over 50% of test sequences have ambiguous amino acids. Low *et al. *[16] subsequently proposed that reliable models should achieve Se(DM) ≥ 0.85 on test sets, a level equivalent to the concordance of co-receptor phenotype assays. Though our SVM model does achieve this threshold, the overall methodology suffers from an important drawback that limits its applicability: all V3 sequences, which may be either from majority or minority HIV-1 viral species, must consist of exactly 35 amino acids (i.e., no indels) that are selected from the standard 20-letter protein alphabet, without ambiguities.

The PDB structure 1ce4 was determined using NMR techniques and consists of 20 models, with model 1 representing the best conformer (lowest target energy function). In order to assess the impact of the conformation on the results, we repeated our analysis by generating an analogous training set based on the use of model 20 and obtaining 10-fold CV performance values. The vast majority of the values obtained using both conformations were within two percentage points of each other, suggesting consistency across all conformers (model numbering corresponds to target function rankings). In particular, Se(DM) measures are identical using both models in the case of RF as well as SVM, while for BDT and NN they differ by one (0.81 vs. 0.80) and two (0.82 vs. 0.80) percentage points, respectively. Additionally, we evaluated contribution of structure information to performance by representing V3 loop sequences in the training set simply based on 4-mer consecutive string clusters. A sliding window of size four over the 35-residue V3 sequence generates 32 such overlapping 4-mer clusters, each of which is represented as a 20-dimensional vector of amino acid counts, and they are appended to form a 640-dimensional vector for each V3 sequence. This sequence-based training set yielded Se(DM) values in the 0.70 - 0.73 range (compared to 0.80 - 0.85 with our structure-based approach), and a paired *t*-test using this data confirmed a statistically significant improvement in performance due to the structural component (*p *< 0.001).

**Figure 5:**
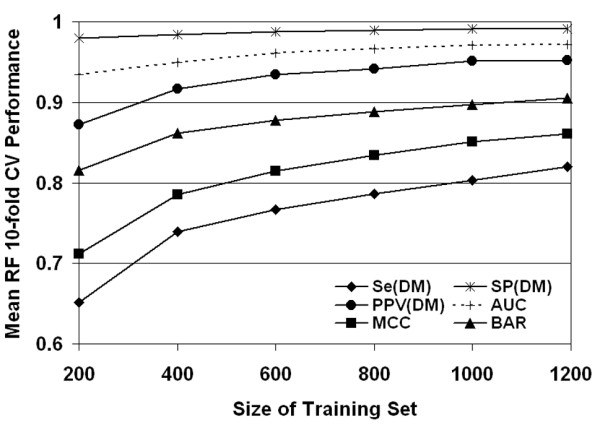
**Learning curves**. Learning curves based on the 10-fold CV performance of the RF algorithm on stratified subsets of increasing size randomly selected from the original dataset. Ten subsets are generated at each size interval, and the average of their performance measures is reported. The availability of additional training data clearly improves performance

Next, learning curves were plotted in order to assess the influence of dataset size on performance, using the RF algorithm as an example (Fig. 5). We generated ten stratified random samples each consisting of 200 variant V3 sequences, where each sample was selected from among the full dataset of 1193 sequences, and mean 10-fold CV performance measures were calculated. Subsequent iterations involved incrementing by 200 mutants the sizes of the ten sampled datasets. The curves clearly reflect that the availability of larger datasets for training leads to significant improvements in RF algorithm performance. With the current dataset size, the learning curves have not reached plateaus, which suggests that additional sequences will further improve performance. This increase in performance may be due to overcoming implicit confounding effects of clade and phylogenetic relatedness that may currently exist in the dataset.

Finally, we attempted to rank the relative importance of the variant V3 loop feature vector components (environmental perturbations at each of the 35 positions in the residual profiles) based on their respective contributions to prediction performance. An SVM classifier was used to rank the attributes by the square of the weights assigned by the SVM, based on the results of a 10-fold CV attribute selection mode. The top ten positions are highlighted by red arrows in Fig. 2 and include positions 11 and 25.

## Conclusions

Variant V3 loop feature vectors generated by the combined sequence-structure *in silico *mutagenesis methodology described in this manuscript have been shown to encode signals that robustly discriminate between the R5 and DM classes, yielding universally reliable predictive models based on a variety of supervised classification machine learning algorithms. Simplicity is a key ingredient to our approach that takes only variant V3 sequence as input and provides instantaneous predictions, whereas the technique of Sander *et al. *[23] involves the modeling of mutant structures, which is expensive, cumbersome, and cannot be completely automated. On the other hand, our methodology is restricted to only 35-residue V3 sequences and cannot accommodate indels or non-standard amino acids, which the Sander *et al. *method is capable of processing. Our models display a modest yet vital improvement in Se(DM) values relative to other published methods. For the SVM classifier in particular, Se(DM) reaches the critical reliability threshold of 0.85 ascertained by Low *et al. *[16]; hence, within the limitations imposed by our approach, this SVM model is capable of correctly determining HIV-1 co-receptor tropism in a timely, inexpensive manner relative to phenotype assays.

## Authors' contributions

IV conceived of the project and supervised the work. MM collected the V3 sequences, generated the dataset, trained the models, performed the statistical analyses, and wrote the first draft of the manuscript. IV and MM participated in editing the text and approved the final manuscript.
